# Long-Lasting Insecticidal Net Coverage and Utilization in Burla Town of Sambalpur District: A Cross-Sectional Study

**DOI:** 10.7759/cureus.61947

**Published:** 2024-06-08

**Authors:** Sanjeeb K Mishra, Gourahari Pradhan, Soumya R Patra, Ashok K Panigrahi, Subrat K Pradhan

**Affiliations:** 1 Field Epidemiology Training Program, Indian Council of Medical Research, Chennai, IND; 2 Community Medicine, Veer Surendra Sai Institute of Medical Sciences and Research, Sambalpur, IND; 3 Pulmonary Medicine, Veer Surendra Sai Institute of Medical Sciences and Research, Sambalpur, IND; 4 General Medicine, Government Medical College, Sundargarh, Sundargarh, IND; 5 Pharmacology, Veer Surendra Sai Institute of Medical Sciences and Research, Sambalpur, IND

**Keywords:** healthcare surveys, long-lasting insecticidal nets, vector-borne diseases, llin use, services utilization, insecticide-treated bed nets

## Abstract

Background

Vector-borne diseases continue to significantly contribute to mortality and morbidity, especially in developing nations. Vector management is a key pillar in combating these diseases, and long-lasting insecticidal nets (LLINs) are cost-effective tools. The Government of India, under the National Vector Borne Disease Control Programme (NVBDCP), has distributed LLINs for free to increase coverage and utilization. This study aims to estimate the coverage and utilization of LLINs in Burla town.

Method

This cross-sectional study was conducted from October to December 2022 in Burla town of Sambalpur in Odisha, India. The estimated sample size was 510 households, assuming 50% coverage. Multi-stage cluster sampling was adopted to select the Anganwadi centers and households. A pretested questionnaire was utilized for data collection by trained personnel through Epicollect5 (Centre for Genomic Pathogen Surveillance, Oxford, UK). Logistic regression was used to identify predictors for LLIN usage.

Results

The survey covered 516 households with 2,541 individuals and 1,165 nets. Household-level coverage was 94.2%, and regular utilization was 45.74%. Skin reactions (35.7%) were the most common reason for non-usage, followed by low mosquito density (12%). Logistic regression showed that the number of rooms (adjusted odds ratio (AOR) = 0.663, p = 0.012), number of bed nets (AOR = 2.757, p < 0.001), knowledge of malaria (AOR = 2.92, p = 0.04), adopting other measures for mosquito control (AOR = 0.295, p < 0.001), and washing the net (AOR = 1.92, p = 0.028) significantly predicted sleeping under mosquito net.

Conclusion

Our study has depicted high coverage of LLINs in Burla town, but utilization needs further improvement. Counseling regarding proper use can decrease the skin reactions responsible for non-usage. Regular health education programs are required to emphasize the benefits of LLIN use, along with regular monitoring and supervision.

## Introduction

About one-fifth of all infections are vector-related diseases accounting for more than 700,000 deaths across the globe each year [1]. Mosquitoes are responsible for the majority of these cases [2]. Malaria is a parasitic infection caused by plasmodium species transmitted by female anopheline mosquitoes [1]. Dengue, a remerging disease, is a viral infection caused by the dengue virus (Flavivirus) transmitted by Aedes mosquitoes. Nearly four billion people in over a hundred countries are at risk of being affected by dengue, with nearly 100 million cases and an estimated 40,000 deaths every year [3]. Many vector-borne diseases are preventable, through protective measures, and community mobilization [1,2].

Integrated vector management is a key strategy that includes indoor residual spraying (IRS) in selected high-risk areas, insecticidal nets (ITNs) in high malaria endemic areas, use of larvivorous fish, anti-larval measures in urban areas, including bio-larvicides and minor environmental engineering, and source reduction for prevention of breeding. The WHO recommends widespread implementation of ITNs. Data from cluster randomized trials support the current policy recommendation for ITNs carried out between 1988 and 2013 that demonstrate the value of ITNs for public health [4].

Long-lasting insecticidal net (LLIN) is an advanced ITN that demonstrates sufficient entomological efficacy even after 20 washes conducted in a laboratory setting. In India, the government procured more than 47 million LLINs that were distributed during 2019-2020 to high-burden areas [5]. The use of LLINs is stated to be highly accepted by the community at large and has contributed to the drastic decline of malaria cases in the country [6].

With financial support from the World Bank and the governments of India and Odisha, anti-malaria activities have been accelerated to improve the delivery of services, especially in remote and inaccessible pockets [7]. The state has made significant progress in controlling malaria with a 90% reduction in the number of cases over the last five years due to targeted interventions [8]. The rate of reduction of vector-borne disease between 2018 and 2019 was 40% against the national average of 17% during the same period [8]. With active interventions, the annual parasite index (API) decreased to less than one per 1,000 population in 23 districts in 2020 compared to eight districts in 2016. Due to the rapid reduction in the positivity rate, the WHO has recorded the Odisha model as a best practice in its World Malaria Report of 2020 [9]. This was made possible due to integrated interventions like the state-led program called Durgama Anchlare Malaria Nirakarana (DAMaN). Around 2.8 crore people were protected from malaria with a distribution of 1.57 crore LLINs under DAMaN. Around 48,455 Accredited Social Health Activist (ASHA) workers were trained and engaged in the diagnosis and management of cases [10].

Studies on LLIN coverage and its ownership in Odisha are scarce. With this background, our study aims to estimate the LLIN coverage and its utilization in the Burla town of Sambalpur district in Odisha, India.

## Materials and methods

This cross-sectional study was conducted in Burla town of Sambalpur district in Odisha state of India. Burla comprises more than 46,000 inhabitants, most of whom live in slums.

Sample size and sampling

Assuming that 50% of households owned at least one LLIN and using a precision of 5%, a confidence level of 95%, a non-response rate of 10%, and a design effect of 1.2, the estimated sample was 506 households. Multi-stage cluster sampling was followed. Anganwadi centers (AWCs) were the initial sampling units that were selected by arranging the 53 AWCs in alphabetical order and selecting 10 of them by random sampling with the help of a random number table. In each AWC area, 51 households were selected by random walk method.

Study design and procedures

A cross-sectional household survey study to estimate the coverage and assess the knowledge, attitude, and practice (KAP) was conducted just after the monsoon (rainy) season, between 1 October 2022 and 3 December 2022. In Sambalpur, two mass distributions of LLINs have taken place since its inception: first in 2016-2017 and second in 2020. The data collection was done by trained data collectors with the help of Epicollect5 (Centre for Genomic Pathogen Surveillance, Oxford, UK). Data collection and management interviews were conducted using structured questionnaires with household heads or, in their absence, with the partner or an adult permanently living in the house. The respondent was explained about the research project in detail and verbal consent was obtained before administering the questionnaire. Households were excluded from the study if the head of the household was not willing to participate or if no adults were available to answer the questionnaire after two separate visits. Interviews included questions on household characteristics, demographic information of all household members like education, occupation, income, number of family members (residents and long-term visitors sleeping in the same house), LLIN ownership, use of LLINs, alternative mosquito control measures, knowledge about diseases caused by mosquito and behavioral questions related to prevention from mosquito-borne diseases. The tool was tested for internal consistency and Cronbach's alpha was 0.67. The questionnaire also enquired about reasons for the non-usage of bed nets by the non-users.

**Figure 1:**
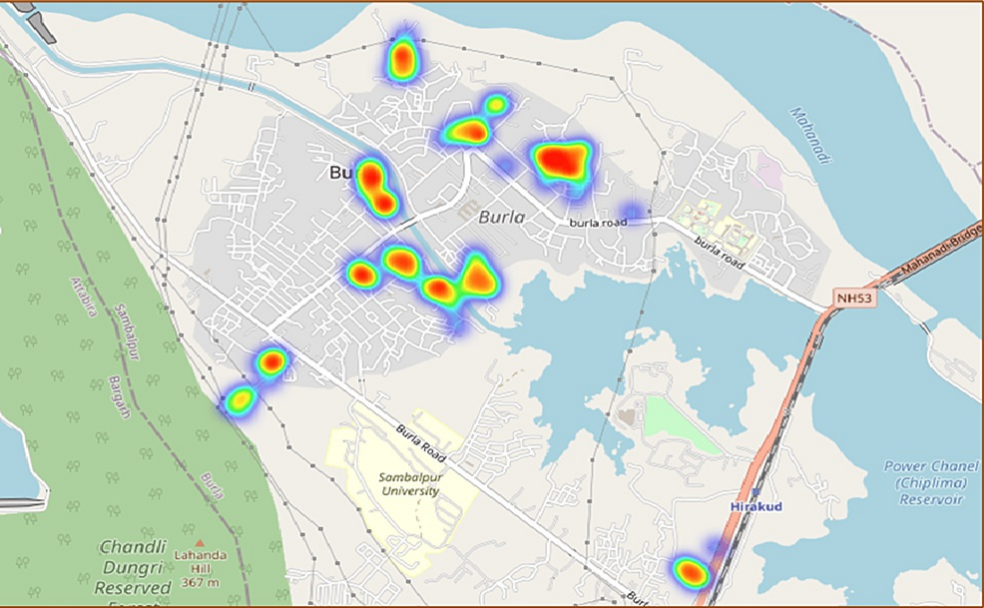
Heat map of the area showing the surveyed clusters in Burla, Sambalpur, Odisha, 2022 (n = 516).

The data collection tool recorded the location of the households, which is shown in Figure 1. The tool also has a provision for pictures of the net in the household with the additional consent of the respondent. Data collected through Epicollect5 was cleaned and analyzed using Epi Info (Centers for Disease Control and Prevention, Atlanta, GA). Categorical variables were expressed in frequency and percentages. Continuous variables were expressed in mean ± standard deviation. The association of variables with the usage of LLIN was tested using the chi-square test after the categorization of continuous variables. To identify the predictors for the usage of bed nets, binary logistic regression analysis was carried out. Analysis results are presented as odds ratios (ORs) with 95% confidence intervals. Approval was obtained before data collection from the Institutional Ethics Committee of Veer Surendra Sai Institute of Medical Sciences and Research.

## Results

**Figure 2:**
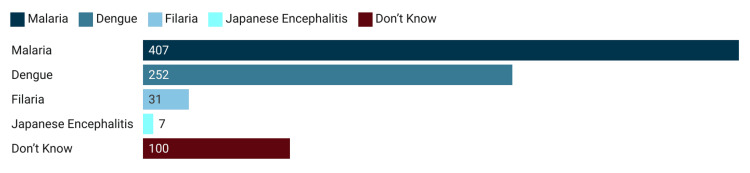
Knowledge of participants on diseases prevented by the mosquito net in Burla, Sambalpur, Odisha, 2022 (n = 516).

The survey covered 516 households with 2,541 individuals and 1,165 nets. Among the surveyed households, 94.2% owned at least one bed net. About 1,107 (95%) of the nets in the households were LLIN supplied under the government scheme. Among the respondents, 364 (70.5%) were females. The age of the respondents ranged from 18 to 90 years, with a mean age of 41.89 (±14.6). The monthly family income of the respondents ranged from 3,000 to 500,000 with a mean of 16.99 thousand. Family size ranged from one to 26, with a mean of 4.92 and a standard deviation of 2.42. Person per room ranged from 0.25 to 11, with a mean of 2.04 ± 1.31. The mean number of nets (treated and untreated) owned per household was 2.26 with a standard deviation of 1.19. As far as the educational status of the head of the family is concerned, nearly 13.76% had no formal education, with a further 42.45% educated below 10th standard. As per the modified Kuppuswamy socioeconomic status scale, 236 households belonged to the lower class, 114 to the upper lower class, 125 to the lower middle class, 31 to the upper middle class, and 10 belonged to the upper class. About 90.3% of the surveyed households obtained nets during the free mass distribution campaign by the Ministry of Health and Family Welfare under the National Vector Borne Disease Control Programme. However, 364 (70.54%) of the households reported that the nets were not supplied to their houses rather they had to collect them from the health center. More than three-fourths of the households had two or more bed nets, six being the highest number of LLINs per family. More than 81% of the households washed the supplied bed nets before use. As far as knowledge about the benefits of mosquito nets is concerned, about 407 (78.88%) correctly identified protection from malaria, followed by dengue (n = 252, 48.84%), filaria (n = 31, 6.01%), and Japanese encephalitis (n = 7, 1.36%) (Figure 2).

Interestingly, only five (0.96%) of the respondents correctly answered all four diseases, and 28 (5.4%) identified three diseases. One hundred (19.4%) of the respondents failed to identify even a single mosquito-borne disease from the list (Figure 2). Only about 29 (5.62%) of the respondents could recall any of their family members suffering from the above-mentioned diseases in their lifetime. The socio-demographics are detailed in Table 1.

**Table 1:** Characteristics of study participants (2022) in Burla, Sambalpur, Odisha, India (n = 516). The modified Kuppuswamy socioeconomic scale was used to assess socioeconomic status.

Variables	Categories	n	%
Age (in years)	≤20	27	5.2
	21-40	236	45.7
	41-60	212	41.1
	≥61	41	7.9
Gender	Female	364	70.5
	Male	152	29.5
Socio-economic status	Lower	319	61.8
	Upper lower	31	6.0
	Lower middle	125	24.2
	Upper middle	31	6.0
	Upper	10	1.9
Education	No education	71	13.8
	Primary	91	17.6
	Secondary	128	24.8
	Matriculate	148	28.7
	12^th^ and above	78	15.1
Number of family members	Four or less	275	53.3
	Five or more	241	46.7
Number of rooms	≤ Two	239	46.3
	Three to four	225	43.6
	≥ Five	52	10.1
Person per room	≤ One	121	23.4
	More than one to two	234	45.3
	≥ Two	161	31.3
Received long-lasting insecticidal nets	Yes	466	90.3
	No	50	9.7
Number of mosquito nets	Zero	30	5.8
	One to two	311	60.3
	Three or more	175	33.9
Washed the supplied net (466)	Yes	421	90.3
	No	45	9.7
Has any family member suffered from malaria, dengue, filariasis, or Japanese encephalitis	Yes	29	5.6
	No	487	94.4
Currently using mosquito net during sleeping	Yes	236	45.7
	No	280	54.3

**Figure 3:**
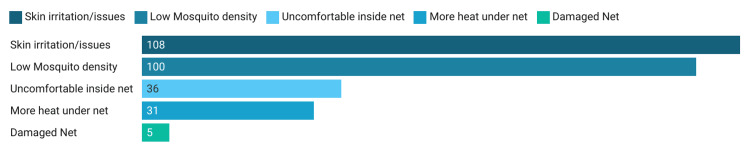
Reasons for non-usage of mosquito nets in Burla, Sambalpur, Odisha, 2022 (n = 280).

Usage of the LLIN was the main outcome of interest and about 236 (45.74%) of the respondents said they were using the nets regularly and slept under it on the previous night also. Nearly four-fifths (n = 429, 83.63%) of the respondents reported having used the supplied bed net at least once. Among the total households, 492 had children aged less than five years and about 104 (21.14%) of the households were using LLIN for their children as a regular practice. Among the non-users (n = 280), the reasons were skin reaction (n = 108, 38.57%), low mosquito density (n = 100, 35.71%), sleeping issues under the net (n = 36, 12.86%), increased heat inside the net (n = 31, 11.07%), and damaged net (n = 5, 1.78%) (Figure 3).

Additional/other measures were utilized by 411 (79.65%) of the households to keep mosquitoes away. These include vaporizer or *Kachua Agarbati* (231), closing doors and windows in the evening (127), burning egg cartons/cow dung (60), and using the net in the door and windows (34).

On the test for association with the chi-square test (on cross-tabulation), the number of rooms (p = 0.024), receiving mosquito nets (p = 0.019), number of bed nets per household (p = 0.000), washing the net (p = 0.000), and adopting other measures to keep mosquito away (p = 0.000) were found to be significantly associated with LLIN usage at an α of 0.05 (Table 2).

**Table 2:** Test of association (chi-square test), 2022-23, in Burla, Sambalpur, Odisha, India (n = 516).

Variables	Categories	User, n (%)	Non-user, n (%)	P-value
Age	≤20	7 (25.9)	20 (71.1)	0.138
	21-40	108 (45.8)	128 (54.2)	
	41-60	104 (49.1)	108 (50.9)	
	≥61	17 (41.5)	24 (58.5)	
Gender	Female	174 (47.8)	190 (52.2)	0.145
	Male	62 (40.8)	90 (59.2)	
Socio-economic status	Lower	149 (46.7)	170 (53.3)	0.455
	Upper lower	17 (54.8)	14 (45.2)	
	Lower middle	55 (44%)	70 (56.0)	
	Upper middle	10 (32.3)	21 (67.7)	
	Upper	5 (50.0)	5 (50.0)	
Education	No education	39 (54.9)	32 (45.1)	0.282
	Primary	41 (45.1)	50 (54.9)	
	Secondary	50 (39.1)	78 (60.9)	
	Matriculate	71 (48.0)	77 (52.0)	
	12^th^ and above	35 (44.9)	43 (55.1)	
Number of family members	Four or less	131 (47.6)	144 (52.4)	0.355
	Five or more	105 (43.6)	136 (56.4)	
Received long-lasting insecticidal nets	Yes	221 (47.4)	245 (52.6)	0.019
	No	15 (30.0)	35 (70.0)	
Number of rooms	≤ Two	124 (51.9)	115 (48.1)	0.024
	Three to four	89 (39.6)	136 (60.4)	
	≥ Five	10 (40.0)	15 (60.0)	
Number of mosquito nets	Zero	0 (0)	30 (100)	0.000
	One to two	141 (45.3)	170 (54.7)	
	Three or more	95 (54.3)	80 (45.7)	
Adopt other measures to keep mosquitoes away	Yes	165 (40.2)	245 (59.8)	0.000
	No	71 (67.0)	35 (33.0)	
Washed the supplied net	Yes	210 (49.9)	211 (50.1)	0.000
	No	26 (27.4)	69 (72.6)	
Any family member suffered from malaria, dengue, filariasis, or Japanese encephalitis	Yes	10 (34.5)	19 (65.5)	0.211
	No	226 (46.4)	261 (53.6)	

Multivariate logistic regression was used to identify predictors of net usage. The variables with p-values less than 0.1 were included in the regression analysis. The enter method was utilized for analysis. The omnibus test showed that the model was significant with an R2 of 0.192 and a correct prediction of 65% of cases. On logistic regression, the number of rooms (adjusted odds ratio (AOR) = 0.663, p = 0.012), number of bed nets (AOR = 2.757, p < 0.001), knowledge of malaria (AOR = 2.920, p = 0.04), adopting other measures for mosquito control (AOR = 0.295, p < 0.001), and washing the net (AOR = 1.920, p = 0.028) significantly predicted sleeping under the mosquito net (Table 3).

**Table 3:** Logistic regression table, 2022-23, Burla, Sambalpur, Odisha, India (n = 516). LLIN: long-lasting insecticidal net; AOR: adjusted odds ratio; SE: standard error.

Variables	B	SE	AOR-Exp B (95% CI)	P-value
Received LLIN	0.21	0.40	1.234 (0.569, 2.780)	0.600
Number of rooms	-0.457	0.17	0.663 (0.450, 0.891)	0.012
Number of mosquito nets	0.72	0.20	2.757 (1.389, 3.039)	0.000
Adopt other measures to keep mosquitoes away	-1.18	0.25	0.295 (0.189, 0.505)	0.000
Washed the supplied net	0.58	0.30	1.920 (0.994, 3.205)	0.028
LLIN helps prevent malaria	0.80	0.25	2.920 (1.368, 3.623)	0.004
Any family member suffered from malaria, dengue, filariasis, or Japanese encephalitis	0.402	0.44	1.494 (0.628, 3.558)	0.364
Constant	0.742	0.53	2.099	0.160

## Discussion

The findings of this study highlight a high level of mosquito net ownership in the surveyed households, with 94.2% of households owning at least one net. A significant proportion of these nets, approximately 95%, were supplied under the government scheme, reflecting the success of government-led initiatives to increase LLIN distribution. These results are in line with previous research on LLIN ownership in regions with active government interventions in northeastern states and other parts of the world [11,12]. The high ownership rates can be attributed to nationwide programs aimed at combatting vector-borne diseases such as malaria and dengue [7].

A study from the high-burden region in Chhattisgarh reported 86% coverage, whereas Bihar reported 81% coverage in another study [13,14]. A study from West Bengal reported 100% coverage of LLIN whereas another from Purulia reported 96% coverage [15,16]. All these studies demonstrate the relatively high coverage of LLIN and demonstrate the efficacy of the government initiatives. However, a significant portion (70.54%) noted that they had to collect the nets from a health center, highlighting potential logistical challenges in the distribution process. The fact that over 81% of households washed the supplied bed nets before use indicates the transmission of government messages before LLIN use. Similar findings were reported by Prakash et al. in North India [11].

The usage of LLINs emerged as a significant area of interest. While approximately 45.74% of respondents reported regular LLIN usage and stated that they slept under the nets on the previous night and similar results were obtained by Millat-Martínez et al. [17]. However, higher usage from Chhattisgarh and West Bengal, and lower usage from Bihar were reported by other studies [13-15]. About 84% reported using the supplied bed nets at least once similar to the results reported by Millat-Martínez et al. [17]. Among households with children under five years old, about 21.14% practiced regular LLIN usage for their children similar to the findings by Millat-Martínez et al. at 19.9% [17].

The awareness of the benefits of mosquito nets was also evaluated. While approximately 78.88% correctly identified protection from malaria as a key benefit, awareness of other mosquito-borne diseases such as dengue, filaria, and Japanese encephalitis varied. These findings are similar to the study by Shrivathsa et al. in Mangaluru who reported 88% knowledge regarding malaria protection and Dey et al. in West Bengal [15,18]. Only a small fraction of respondents correctly identified all four diseases. This highlights the need for more comprehensive health education programs.

Skin reactions, low mosquito density, sleeping issues, heat inside the net, and damaged nets were identified to be common reasons for non-usage. Similar issues were also highlighted by other studies from this region [14,18]. Additional measures to repel mosquitoes were also employed by a substantial portion of the households, with vaporizers, closing doors and windows, burning egg cartons/cow dung, and using nets on doors and windows being popular choices.

The analysis of factors associated with LLIN usage revealed some interesting insights. Several persons per room, receiving a mosquito net, the number of bed nets per household, and washing the nets were all significantly associated with LLIN usage. These results suggest that household characteristics, availability of nets, and maintenance practices play a vital role in determining LLIN usage. Other studies also depicted similar associations [4,13,17].

Strength and limitations

The study is one of the few to provide these estimates and also provides an evaluation of the national program on vector-borne diseases in Odisha. The adopted questionnaire had a Cronbach's alpha of 0.67, which is of moderate to low internal consistency and may well have resulted from a limited number of items. The small geographical region may also be a limitation for the generalizability of the results.

## Conclusions

To summarize, our study provides a comprehensive picture of LLIN coverage and ownership in the surveyed households. The high ownership rates and government interventions have contributed to widespread access to LLINs. The reasons for non-usage, such as skin reactions and discomfort, call for improved LLIN design and health education programs. Understanding the factors associated with LLIN usage is crucial for targeting interventions and ensuring the effectiveness of LLIN distribution programs. Further research, particularly through qualitative methods, can shed more light on the predictors of LLIN usage, facilitating evidence-based interventions to combat vector-borne diseases.
